# A Systematic Review of the Learning Curves of Novices and Trainees to Achieve Proficiency in Laparoscopic Skills: Virtual Reality Simulator Versus Box Trainer

**DOI:** 10.7759/cureus.72923

**Published:** 2024-11-03

**Authors:** Dharshanan Raj Selva Raj, Shaarven Kumar, Kasthuri Nallathamby, Kisshan Raj, Marta Hristova

**Affiliations:** 1 Plastic Surgery, Aberdeen Royal Infirmary, Aberdeen, GBR; 2 Internal Medicine, Stepping Hill Hospital, Manchester, GBR; 3 Medicine, Whiston Hospital, Mersey, GBR; 4 Urology, Wrexham Maelor Hospital, Wrexham, GBR; 5 Stroke, Whiston Hospital, Liverpool, GBR

**Keywords:** box trainers, laparoscopic simulator, laparoscopic skills, simulation trainer, virtual reality in medical education, virtual reality (vr)

## Abstract

Laparoscopic surgery, established in the 1980s, has become a primary treatment method across various surgical specialities due to its advantages over open surgery, including shorter recovery times and fewer complications. Mastery of laparoscopic skills is essential for novice and junior trainees, who must develop hand-eye coordination, depth perception, and instrument handling. This systematic review examines the learning curves of novices using box trainers compared to those using virtual reality (VR) simulators to attain proficiency in laparoscopic skills. The learning curves are assessed through metrics such as the Objective Structured Assessment of Technical Skills (OSATS), the Global Operative Assessment of Laparoscopic Skills (GOALS), and the Global Rating Scale (GRS) based on the time taken to complete specific tasks. A systematic review was conducted to examine the learning curves of novices in achieving proficiency in laparoscopic skills. An extensive literature search on PubMed, Embase, and Cochrane was conducted using keywords like laparoscopic surgery, minimally invasive surgery (MIS), virtual reality (VR), box trainers, and simulation. Thirteen articles were reviewed and analysed in this study. The analysis of data from these papers, along with subsequent meta-analyses, revealed no significant differences in the learning curves of trainees using VR simulators compared to those using box trainers. Variability in conclusions among studies arose from differences in assessment methods and parameters. Overall, there is no consensus on which training modality yields a steeper learning curve. The systematic review and meta-analysis of 13 studies concluded that there was no notable difference in the learning curves of trainees using VR simulators compared to those using box trainers. It suggests the need for standardized assessment methods and parameters across studies. Generally, box trainers are preferred for mastering core laparoscopic skills like knot tying and suturing, while VR simulators are more effective for teaching specific surgical procedures, such as cholecystectomy and hemicolectomy.

## Introduction and background

Laparoscopic surgery has revolutionized modern surgical therapies due to the advantages it offers, including reduced surgical timing, reduced pain and reduced scarring, shorter recovery timing, and overall reduced post-operative side effects [1]. Specialities such as general surgery, urology, obstetrics, and gynaecology are just some of the surgical specialities that have adopted the use of laparoscopy, further defining the standard of care. However, with these advancements come new challenges faced by surgeons such as the lack of depth perception (as images go from 3D to 2D on screen), inverted axis, and the diminished tactile feedback compared to an open surgery [1]. Therefore, it is vital to have an effective simulation and training to overcome these challenges.

It is generally known among surgical trainees that the learning curve for laparoscopic surgeries is longer and more difficult, as evident in complex surgeries such as laparoscopic colorectal surgeries. This is particularly evident in research done by Madan et al., which demonstrates the lack of laparoscopic practice among trainees with the difficulty of overcoming the lack of depth perception and the decreased haptic feedback [2].

Hence, the need for effective training and simulation outside the operating theatre is important in mastering the skill of laparoscopic surgery. Training in the manipulation of endoscopic instruments, depth perception, hand-eye coordination, and handling both the camera and tissue is essential for achieving competency [3]. These can be achieved with the more popular methods available, which would be box trainers and virtual reality (VR) machines.

The objective of this meta-analysis was to assess the learning curve of the two most common modalities available to trainees and novices to achieve proficiency in laparoscopic skills.

## Review

Materials & methods

This systematic review with meta-analysis involves data collected from original research articles and reviews. This study seeks to evaluate the learning curve of beginners and trainees utilizing VR simulators and box trainers, while also comparing their proficiency in laparoscopic tasks and skills.

Relevant articles from 2001 to 2024 were identified and collected via the internet from various sources such as Cochrane, PubMed, and Embase.

**Figure 1:**
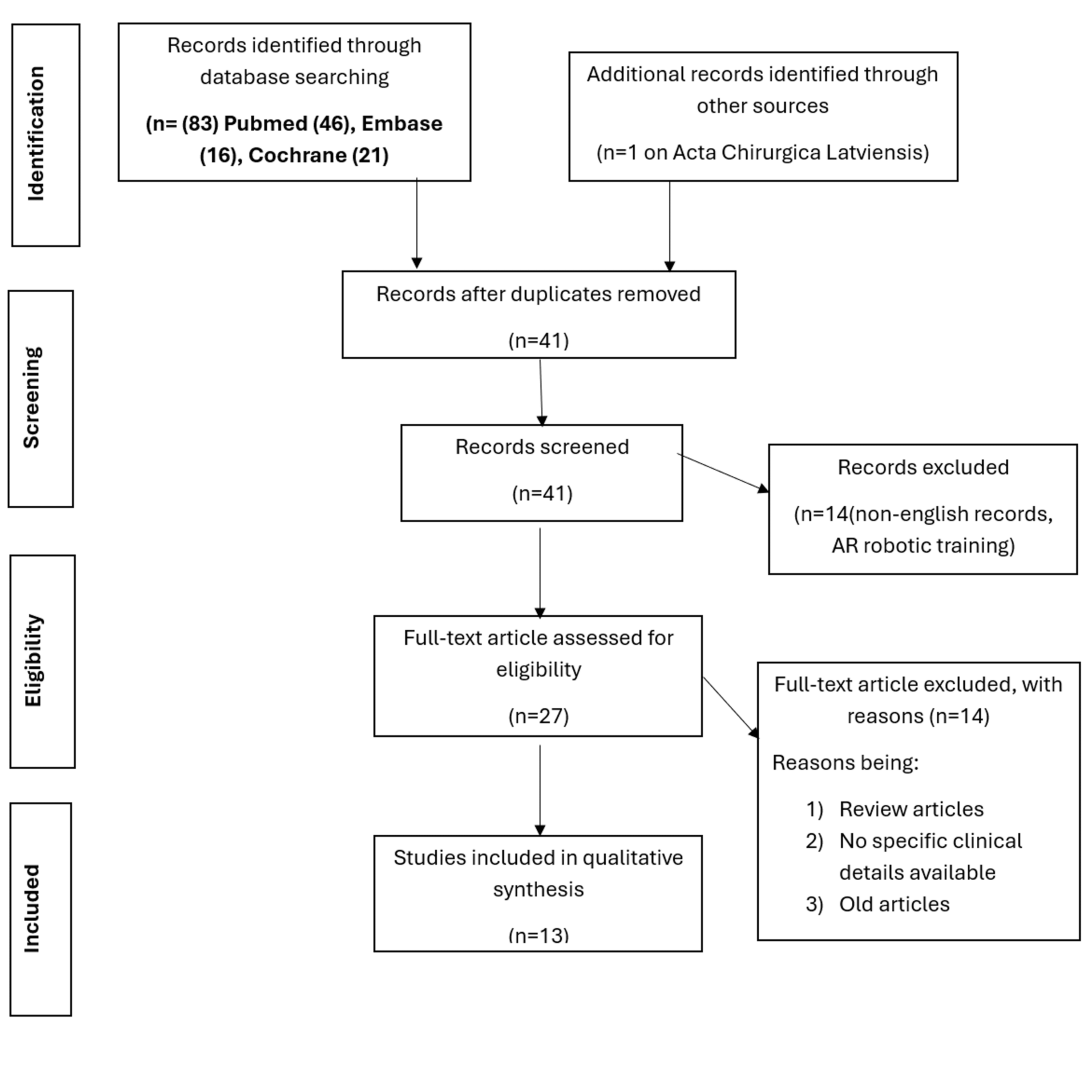
PRISMA chart. This systematic review was done in accordance with the Preferred Reporting Items for Systematic Reviews and Meta-Analyses (PRISMA) guidelines. AR: augmented reality.

EndNote (Clarivate, Philadelphia, PA) was used for data inclusion and citations. Only papers published in English were included. The evidence in this review adhered to the Preferred Reporting Items for Systematic Reviews and Meta-Analyses (PRISMA) guidelines, as illustrated in Figure 1, and was consistent with the recommendations from the Oxford Centre for Evidence-Based Medicine (CEBM) established in 2011. The risk of bias in the included studies was assessed using the ROB2 tool, while the Cochrane guide was used to assess the quality of studies and the risk of bias in non-randomized interventions using the ROBINS-1 tool. The certainty of the included evidence was determined by using the modified version of the Newcastle-Ottawa Scale (NOS). Data collected were then reviewed and analysed using Review Manager (RevMan, Cochrane Collaboration, London, UK).

PICOS Criteria

The study employs the PICOS (population, intervention, control, outcome, and study design) criteria, focusing on a population of medical students, laypeople, surgical trainees, and junior registrars (P). It compares the intervention of a VR simulator (I) against a laparoscopic box trainer (C) in assessing primary outcomes of learning curves in laparoscopic training, as well as secondary outcomes of integrated simulator metrics and error scores (O). The study is structured as a systematic review with meta-analysis (S).

Literature Search Strategy

A total of 84 systematic reviews and original articles were identified from 2001 to 2018 using Embase, Cochrane, and PubMed. The literature search focused exclusively on articles in English and imposed no geographical restrictions. The search utilized terms and keywords such as box trainers, virtual reality (VR), laparoscopic, and novices. Articles were screened based on the inclusion and exclusion criteria outlined below. Keywords used to search the database included “Learning methods AND ‘proficiency in basic laparoscopic skills’” and “effectiveness AND ‘simulation training’”.

MeSH terms such as “virtual reality”, “learning curves”, “laparoscopic surgery”, “novices”, and “simulation training” were also included in the search strategies.

Database Searches

PubMed, Embase, and Cochrane searches were conducted for articles published from 2001 to 2024. Additional resources comprised literature from books found in the Queen Mary University of London library in Whitechapel. Efforts were made to gather information related to this topic from all available published, unpublished, and ongoing studies across various data sources. References from included studies, and the "related articles" function of PubMed, were also reviewed to identify any further literature.

Type of Study (Inclusion and Exclusion Criteria)

The inclusion criteria for the study encompass medical students, doctors, and junior surgical trainees, all with no prior laparoscopic training. The exclusion criteria include senior surgical trainees, senior registrars, consultant surgeons, and participants with a background in laparoscopic training or surgery.

Interventions included VR machines that allow novices and surgical trainees to perform laparoscopic tasks such as tying a knot or simulated cholecystectomy. The learning curves of novices using VR machines and box simulators were compared and analysed.

Learning curves were formulated based on the differences in scores on the Objective Structured Assessment of Technical Skills (OSATS), the Global Operative Assessment of Laparoscopic Skills (GOALS), and the Global Rating Scale (GRS), which were used to assess laparoscopic skills performed via box trainers and VR simulators.

Data Management and Extractions

Data from eligible studies were extracted, which included the name of the first author, year of publication, study design, study location, language, country, risk of bias, and outcomes from the studies. Papers from 2001 to 2024 were included, with no geographical restrictions applied. Only articles published in English were reviewed.

Risk of Bias Assessment

**Figure 2:**
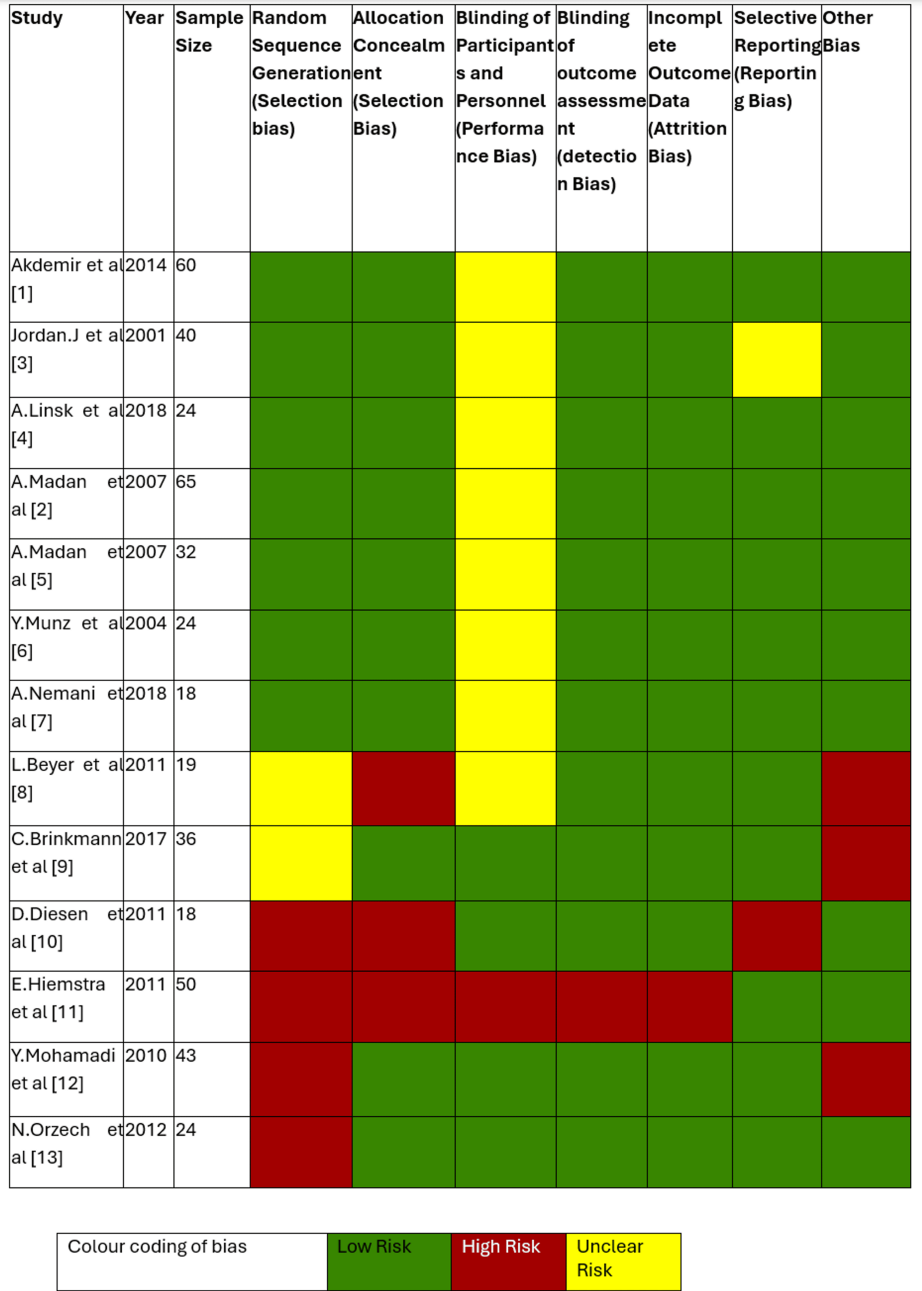
Data collection for risk of bias assessment for the studies included according to the Cochrane Collaboration Risk of Bias tool.

**Figure 3:**
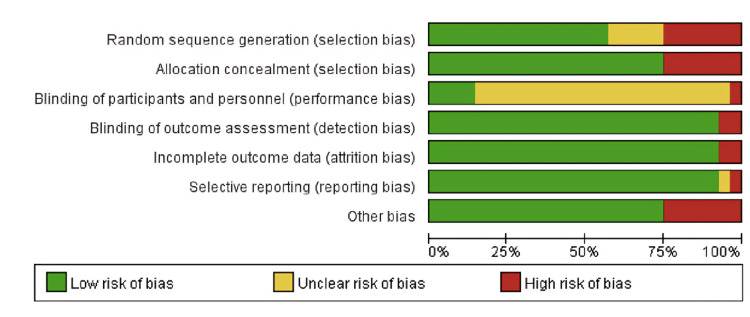
Risk of bias summary.

All studies were reviewed by two independent investigators. Two reviewers assessed the risk of bias according to the recommendations in the Cochrane Handbook for Systematic Reviews of Interventions, using the Cochrane Risk of Bias tool (Cochrane Collaboration, London, UK), as shown in Figures 2, 3. Any discrepancies or disagreements identified were resolved through consensus.

Statistical Analysis

Statistical analysis was conducted following the established protocol and the guidelines outlined in the Cochrane Reviewers Handbook. Data were analysed using RevMan 5.4 software from the Cochrane Collaboration. Results are presented as a mean difference with a 95% confidence interval (CI), and any dichotomous outcomes are presented as an odds ratio (OR) with a 95% CI. Heterogeneity was reviewed using the Cochrane Q statistics, in which p-value <0.10 indicates significant heterogeneity.

Results

The 13 papers included in this systematic review were randomized controlled trials (RCTs). These included a total of 494 participants, all novices with no prior experience in laparoscopy, divided into two groups. The trial group (TG) included 178 participants tested using the VR simulator, while the control group (CG), which included 316 participants, was tested with box trainers.

The assessment methods and main outcomes of the 13 papers were reviewed, which were ultimately used to plot the learning curve of novices using box trainers and VR simulators. Table 1 demonstrates the included studies, along with their comparison, assessment methods, and outcomes.

**Table 1:** Comparison between the included studies. FLS: Fundamentals of Laparoscopic Surgery; VBLaST: Virtual Basic Laparoscopic Skill Trainer; GOALS: Global Operative Assessment of Laparoscopic Skills; OSATS: Objective Structured Assessment of Technical Skills; CUSUM: cumulative summation.

Author	Year of study	Number of participants in the trial group (TG)	Number of participants in the control group (CG)	Repetitions	Assessment methods	Outcomes
Akdemir et al. [1]	2014	20 (no experience)	40 (novices with box trainers)	5 weeks	Live first bilateral tubal ligation surgery	Number of surgical errors
Jordan et al. [3]	2001	8 (no experience)	32	Same time	Laparoscopic pattern cutting task	Number of accurate and inaccurate incisions
Linsk et al. [4]	2018	17 (no experience)	48	3 weeks	PC tasks	Time, error
Madan et al. [5]	2007	18 (no experience)	14	10 sessions	Activities on porcine	Time, error
Munz et al. [6]	2004	8 (no experience)	16	30 minutes per week over 3 weeks	Laparoscopic tasks	Errors, total number of movements
Nemani et al. [7]	2018	6 (no experience)	12	12 days	FLS and VBLaST trials	CUSUM, learning curve
Beyer et al. [8]	2011	6	13	4 months	Live laparoscopic cholecystectomy	GOALS scores
Brinkmann et al. [9]	2017	18	18	5 days	Live laparoscopic cholecystectomy on a pig	GOALS scores
Diesen et al. [10]	2011	10	8	6 months	Laparoscopic exercises on a porcine live model	OSATS scores
Hiemstra et al. [11]	2011	20	30	20 minutes	Pattern cutting	Time taken, correct and incorrect incisions
Madan et al. [2]	2007	17	48	200 minutes	Box training tasks	Time, error
Mohammadi et al. [12]	2010	17	26	6 sessions	Box training tasks	Time taken, accuracy of tasks
Orzech et al. [13]	2012	13	11	Reaching proficiency	Live Nissen fundoplication	Time, OSATS scores

Virtual Reality Simulators Versus Box Trainers

The learning curve of novices using box trainers versus VR simulators was assessed in the 13 papers included in this review. Among these papers, seven showed no differences between the two assessment groups, while the other six had inconclusive or controversial results. Brinkmann et al. concluded that there were no significant differences between novices trained with box trainers and VR simulations but demonstrated that trainees using box trainers subsequently performed better at live laparoscopic cholecystectomies compared to those trained with VR simulations [9]. Hiemstra et al. demonstrated better outcomes among those trained with box trainers, whereas Jordan et al. concluded the opposite, describing that VR simulation training often resulted in better accuracy and precision compared to its counterpart group [3,11]. Mohammadi et al. and Orzech et al. showed no difference between the groups but indicated that the use of VR simulation has the added benefit of increasing interest in learning laparoscopic skills among trainees, as it was more immersive [12,13].

Meta-Analysis

**Figure 4:**
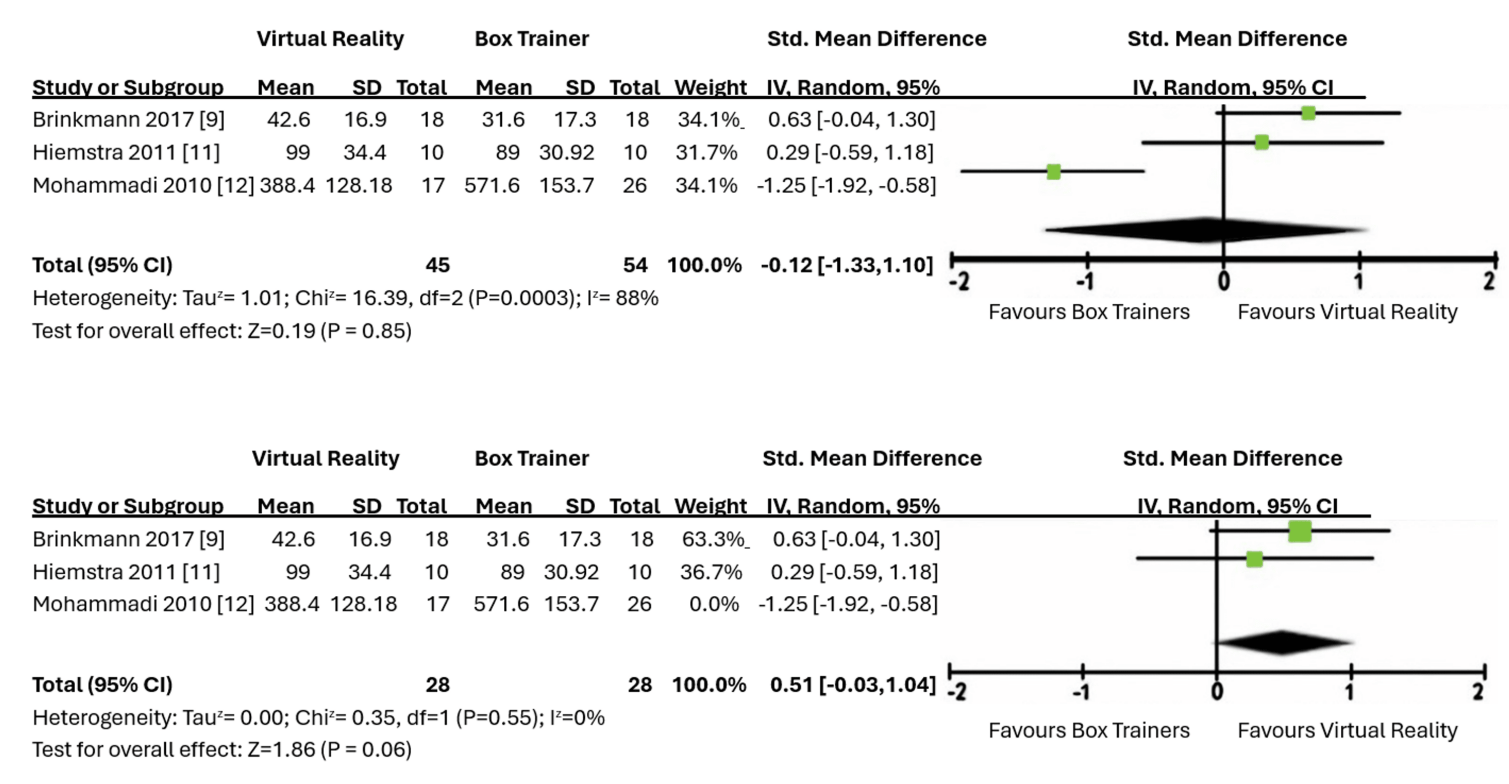
Forest plot of virtual reality (VR) vs. box trainers (time improvement).

This review included a meta-analysis of six of the 13 papers. With two meta-analyses being carried out, both indicated that there was no significant difference between the two groups using VR and box training. As illustrated in Figure 4, the first meta-analysis, which included three papers and assessed improvement over time, showed an estimated mean difference of -0.12 using the random effects model, with a confidence interval between -1.33 and 1.10.

**Figure 5:**
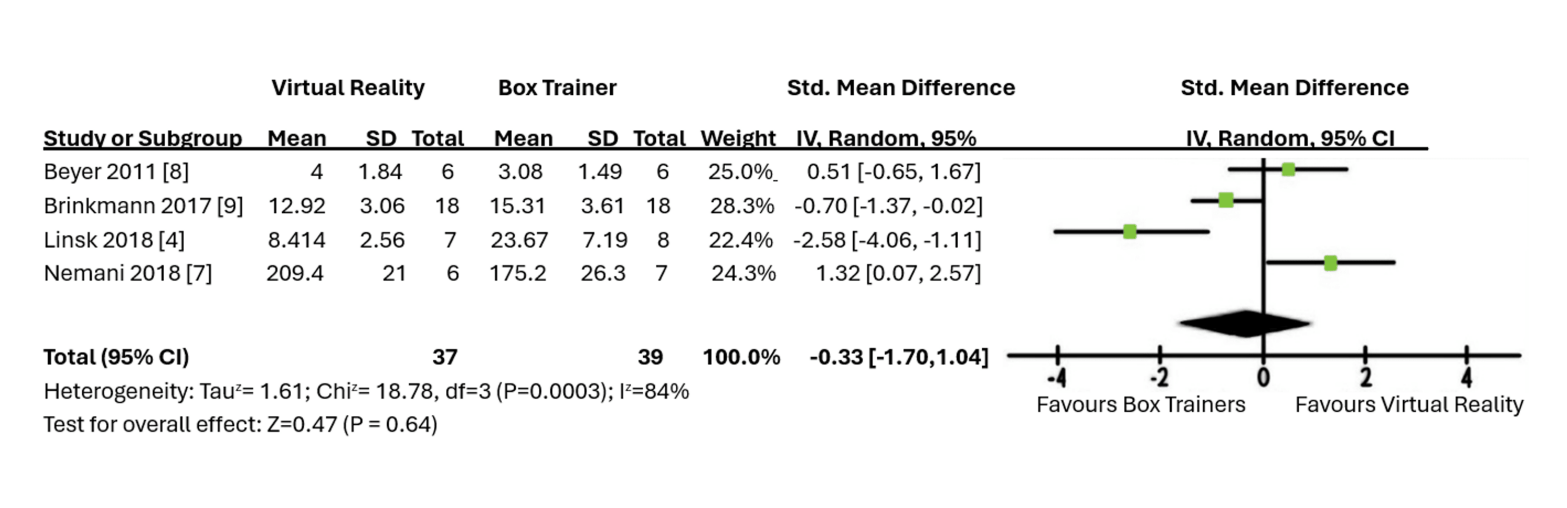
Forest plot of virtual reality (VR) vs. box trainers (score difference and improvement).

A second meta-analysis was performed with another four studies to assess the improvement of scores, as shown in Figure 5. This showed an estimated mean difference of -0.33 using the random effects model, with a confidence interval between -1.70 and 1.04. Hence, a conclusion regarding which learning curve is steeper when comparing box training and VR simulation training could not be made, as there were no significant differences between them.

Both meta-analyses showed a high level of heterogeneity likely due to the differences in training curricula and assessment methods employed in the studies.

Discussion

This systematic review and meta-analysis were done to assess the learning curves of novices regarding laparoscopic skills training with box trainers and VR simulators. Thirteen papers were reviewed to evaluate these two methods by performing different tasks. The overall results displayed no significant difference between the learning curves of both methods of laparoscopic training.

There are, however, a few meta-analyses comparing similar groups that have arrived at different conclusions. This can be demonstrated in the 2019 paper by Guedes et al., which concluded that VR training was significantly superior to box training in terms of time taken and total scores [14]. Alaker et al. demonstrated similar effectiveness in training with VR and box trainers but also showed an improvement in core laparoscopic skills such as knot tying and suturing with the use of box trainers compared to VR training [15].

The 13 papers reviewed assessed the proficiency and competency of novices after training by achieving a set benchmark. These studies, however, did not consider the base laparoscopic skill set of trainees prior to training, which affects the consistency among participants. The different number of repetitions and times were also inconsistent factors among the studies. These are possible explanations for the inability of the 13 papers included to achieve a consensus and for their differing conclusions.

VR simulators are an unparalleled method for mimicking the actual operating theatre environment and possible complications such as bleeding and leaks. This results in a safe environment for trainees to practice surgical procedures before entering the operating theatre. The downside, however, is the cost of the simulator itself, which is the main limiting factor for widespread use. Due to this, it has been suggested that VR simulation can be used to instil interest among medical students and to teach operative steps to junior trainees, but it should not be the only learning tool for them.

Box trainers, on the other hand, are more cost-effective and cheaper to operate. Multiple studies have shown that trainees who trained with box trainers have a better perception of depth and improved hand-eye coordination compared to those trained with VR simulation alone. It has also been demonstrated that trainees using box trainers alone can master knot tying and suturing compared to those using VR simulators. It would be beneficial to investigate the learning curves of trainees using augmented reality (AR) compared to those using box trainers.

Limitation and recommendation

There have been two limitations in this systematic review and meta-analysis. The lack of uniformity in the assessment tools used and the differences in parameters across the 13 reviewed papers have resulted in different conclusions from the authors. This would be the first limitation of this review. Hence, the reason to perform a meta-analysis is to assess the time and score improvement between both groups.

The second limitation would be the high heterogeneity of the primary outcome of this systematic review, which might affect the results.

There are multiple recommendations moving forward. We would recommend having a larger sample size for both groups, with a fixed defined parameter and set assessment tool. Other than this, we would recommend using a hybrid model of learning by utilizing both the box trainers and VR simulators, as this would enable trainees to enhance their depth perception skills and improve hand-eye coordination with the box trainers before moving on to VR simulations to practice the operation steps. Finally, with AR being in the trial stages, it would be valuable to assess the learning curves of VR simulation and AR among trainees to explore what innovations they might bring to laparoscopic training.

## Conclusions

In essence, this systematic review and meta-analysis of 13 papers involving 494 participants evaluated the learning curves of novices in laparoscopic skills training through two primary modalities: VR simulators and box trainers. The analysis yielded no significant differences in the learning curves of trainees when developing expertise in laparoscopic surgery. While some studies have demonstrated an advantage of VR simulators with regard to engagement and immersive experience, others have highlighted the benefits of box trainers in terms of better facilitating depth perception and hand-eye coordination.

The potential of this research to enhance surgical education, a critical aspect of modern healthcare, should not be overlooked. As various specialities embrace the introduction of laparoscopic surgery as the standard of care, it is paramount that surgical trainees are effectively trained to ensure positive patient outcomes and safety. Being able to identify the most effective training modality can lead to improved educational strategies, ultimately resulting in better-trained surgeons. To complement the existing literature, future research should focus on several key areas. Longitudinal studies tracking the progress of trainees over time with regard to skill retention could be an effective way of assessing different training modalities. Research design could include RCTs comparing specific skill sets taught via VR versus box trainers that would help define their relative efficacy and minimize bias. A cost-effective analysis of implementing VR training compared to traditional box trainers would help assess the financial implication of each training method, enabling institutions to make an informed decision. Ultimately, enhancing training strategies is crucial for preparing the next generation of surgeons to meet the demands of modern surgical practices effectively.

## References

[1] Akdemir A, Sendağ F, Oztekin MK (2014). Laparoscopic virtual reality simulator and box trainer in gynecology. Int J Gynaecol Obstet.

[2] Madan AK, Frantzides CT (2007). Prospective randomized controlled trial of laparoscopic trainers for basic laparoscopic skills acquisition. Surg Endosc.

[3] Jordan JA, Gallagher AG, McGuigan J, McClure N (2001). Virtual reality training leads to faster adaptation to the novel psychomotor restrictions encountered by laparoscopic surgeons. Surg Endosc.

[4] Linsk AM, Monden KR, Sankaranarayanan G (2018). Validation of the VBLaST pattern cutting task: a learning curve study. Surg Endosc.

[5] Madan AK, Frantzides CT (2007). Substituting virtual reality trainers for inanimate box trainers does not decrease laparoscopic skills acquisition. JSLS.

[6] Munz Y, Kumar BD, Moorthy K, Bann S, Darzi A (2004). Laparoscopic virtual reality and box trainers: is one superior to the other?. Surg Endosc.

[7] Nemani A, Ahn W, Cooper C, Schwaitzberg S, De S (2018). Convergent validation and transfer of learning studies of a virtual reality-based pattern cutting simulator. Surg Endosc.

[8] Beyer L, Troyer JD, Mancini J, Bladou F, Berdah SV, Karsenty G (2011). Impact of laparoscopy simulator training on the technical skills of future surgeons in the operating room: a prospective study. Am J Surg.

[9] Brinkmann C, Fritz M, Pankratius U (2017). Box- or virtual-reality trainer: which tool results in better transfer of laparoscopic basic skills?—A prospective randomized trial. J Surg Educ.

[10] Diesen DL, Erhunmwunsee L, Bennett KM (2011). Effectiveness of laparoscopic computer simulator versus usage of box trainer for endoscopic surgery training of novices. J Surg Educ.

[11] Hiemstra E, Terveer EM, Chmarra MK, Dankelman J, Jansen FW (2011). Virtual reality in laparoscopic skills training: is haptic feedback replaceable?. Minim Invasive Ther Allied Technol.

[12] Mohammadi Y, Lerner MA, Sethi AS, Sundaram CP (2010). Comparison of laparoscopy training using the box trainer versus the virtual trainer. JSLS.

[13] Orzech N, Palter VN, Reznick RK, Aggarwal R, Grantcharov TP (2012). A comparison of 2 ex vivo training curricula for advanced laparoscopic skills: a randomized controlled trial. Ann Surg.

[14] Guedes HG, Câmara Costa Ferreira ZM, Ribeiro de Sousa Leão L, Souza Montero EF, Otoch JP, Artifon ELA (2019). Virtual reality simulator versus box-trainer to teach minimally invasive procedures: a meta-analysis. Int J Surg.

[15] Alaker M, Wynn GR, Arulampalam T (2016). Virtual reality training in laparoscopic surgery: a systematic review & meta-analysis. Int J Surg.

